# The process evaluation of a citizen science approach to design and implement workplace health promotion programs

**DOI:** 10.1186/s12889-022-14009-8

**Published:** 2022-08-24

**Authors:** Lisa Lelie, Henk F. van der Molen, Mandy van den Berge, Sophie van der Feltz, Allard J. van der Beek, Carel T. J. Hulshof, Karin I. Proper

**Affiliations:** 1 Department of Public and Occupational Health, Amsterdam Public Health Research Institute, Amsterdam UMC, Vrije Universiteit Amsterdam, 1007 MB Amsterdam, The Netherlands; 2 Department of Public and Occupational Health, Amsterdam Public Health Research Institute, Coronel Institute of Occupational Health, Netherlands Center for Occupational Diseases, Amsterdam UMC location AMC, University of Amsterdam, 1100 DD Amsterdam, The Netherlands; 3 Centre for Nutrition, Prevention and Health Services, National Institute for Public Health and the Environment, 3721 MA Bilthoven, The Netherlands

**Keywords:** Blue-collar workers, Workplace health promotion program, Citizen science, Process evaluation

## Abstract

**Background:**

Many workplace health promotion programs (WHPPs) do not reach blue-collar workers. To enhance the fit and reach, a Citizen Science (CS) approach was applied to co-create and implement WHPPs. This study aims to evaluate i) the process of this CS approach and ii) the resulting WHPPs.

**Methods:**

The study was performed in two companies: a construction company and a container terminal company. Data were collected by questionnaires, interviews and logbooks. Using the framework of Nielsen and Randall, process measures were categorized in the intervention, context and mental models. Interviews were transcribed and thematically coded using MaxQDA software.

**Results:**

The involvement in the CS approach and co-creating the WHPPs was positively experienced. Information provision, sustained engagement over time and alignment with the workplace’s culture resulted in barriers in the CS process. As to the resulting WHPPs, involvement and interaction during the intervention sessions were particularly experienced in small groups. The reach was affected by the unfavorable planning off the WHPPs and external events of re-originations and the covid-19 pandemic.

**Discussion:**

Continuous information provision and engagement over time, better alignment with the workplace’s culture and favorable planning are considered to be important factors for facilitating involvement, reach and satisfaction of the workers in a Citizen science approach to design and implement a WHPP. Further studies continuously monitoring the process of WHPPs using the CS approach could be helpful to anticipate on external factors and increase the adaptability.

**Conclusions:**

Workers were satisfied with the involvement in WHPPs. Organizational and social cultural factors were barriers for the CS approach and its reach. Involvement and interaction in WHPPs were particularly experienced in small grouped sessions. Consequently, contextual and personal factors need be considered in the design and implementation of WHPPs with CS approach among blue-collar workers.

## Background

Blue-collar workers have a poorer health and are at higher risk for several chronic diseases compared to white-collar workers [1–3]. This can partly be explained by the fact that blue-collar workers more often work in unhealthy work environments and are more likely to suffer from unhealthy lifestyle behaviors [2–6]. For example, it is known that blue-collar workers often have high physical work demands, low job control, and high job insecurity compared to white-collar workers. Furthermore, blue-collar workers more often have a less healthy diet, are more often overweight, smoke more often and are less physically active during leisure-time [1–3]. This multifactorial problem impacts not only blue-collar workers, but also affects their employers and society as a whole due to the associated loss of productivity and health care costs [7, 8]. To promote health in this population, the implementation of Workplace Health Promotion Programs (WHPPs) is needed [9–11].

Health promotion programs at the workplace have gained interest and have increasingly been implemented in occupational settings [12–14]. The workplace is a promising setting to promote healthy behavior among workers, since they spend a lot of time at the workplace, are working at a certain location, and can therefore be more easily reached [13, 15, 16]. However, when WHPPs are implemented at the workplace, the group of blue-collar workers is often less reached and is more likely to drop out earlier than white-collar workers [16–18]. A misalignment with the needs, skills and capacities of blue-collar workers may be underlying this, as WHPPs are predominantly cognition-focused and therefore not always fit well to the needs and skills of the more often practically oriented blue-collar workers [19, 20].

A participatory approach with active cooperation of the target group is regarded as key element in the development of a WHPP that is suited to the needs and skills of blue-collar workers [21, 22]. Citizen Science is such a participatory method which focuses on active participation of the target group and other important stakeholders in different stages of research, including the design and implementation [23, 24]. In this study the Citizen Science approach consisted of active involvement and co-creation with the target population. The approach was focused on acquiring insights into the needs of workers at both companies to promote health through health interventions.

The Citizen Science approach has been applied for various purposes and in different settings [25, 26]. For example, a Citizen Science approach has been applied in a study focusing on the improvement of a community’s health in a disadvantaged neighborhood [27]. This study showed that Citizen Science could be an effective strategy to engage the community and to develop and implement health promotion projects. Citizen Science therefore offers potential for the use in other settings, including health promotion in the occupational setting.

Despite the flexible implementation possibilities of Citizen Science, limited knowledge is available about the feasibility and effect of Citizen Science in an occupational setting. Citizen Science of this study was adapted to develop and implement WHPPs in collaboration with blue-collar workers to optimize the fit and reach of the WHPPs [28] but it interfered with the Covid-19 pandemic. As this is, to our knowledge, the first study that applied Citizen Science to co-create and implement a WHPP, it is valuable to gain insight into the experiences and the process of the Citizen Science approach and the resulting WHPPs, also with interfering events as a unexpected pandemic situation.

The aim of this study was to evaluate the process of a Citizen Science approach and the resulting WHPPs in two companies with the use of the Nielsen and Randall framework. The research questions that will be addressed are:

1. what are the experiences of the citizen science approach regarding the process components: communication, participation, satisfaction, culture and events?
2. what are the experiences of the implemented resulting interventions regarding the process components: communication, participation, reach, satisfaction, tailoring, exposure, participation/utility, culture, readiness for change and perception?

## Methods

Besides logbooks, data were collected through interviews with workers of both companies and a questionnaire at the construction company at the beginning (T0) and end of the intervention (T1).

### Study design and population

The current process evaluation was part of a study to the application and evaluation of a Citizen Science approach at the workplace to promote blue-collar workers’ health [28]. This approach was applied as a participatory strategy with the aim to develop and implement a health-promoting WHPP that was tailored to the needs, possibilities and skills of the target population.

The study population consisted of blue-collar workers, working in 1) a construction company and 2) a container terminal company. Since the interventions were implemented company-wide, a small number of white-collar workers was also invited – as requested by the management of both companies - including supervisors and office workers. The construction company is specialized in building, renovation, restoration, and maintenance services. In total, the company has 30 blue-collar workers who work internally at the carpentry factory, 103 blue-collar workers who work at temporary construction sites, and 176 white-collar workers with an office function. The container terminal company, has 445 blue-collar workers and 170 white-collar workers. This company is engaged in shipping and landside services, including transport, storage, and maintenance services for containers, gate and reefer services.

Human resource (HR) advisors, managers or prevention workers at both companies invited workers to participate in the intervention, to complete the questionnaire and to participate in the evaluation interviews. If desired, workers were able to actively participate as citizen scientists. In the context of this study, citizen scientist were the intervention’s ambassadors thereby contributing to the promotion of the intervention, motivation of colleagues and division of activities.

The Medical Ethical Committee of the VU University Center (Amsterdam, the Netherlands) concluded that approval of the study protocol was not required for conducting this study (reference number: 2018.138). In advance of the study engagement, all included participants signed an informed consent.

### Citizen science approach

The participatory nature of the Citizen Science approach consisted of active involvement and co-creation with the target population. This approach focused on acquiring insights into the needs of workers regarding health improvement and possibilities at both companies to promote health through health interventions. For the implementation of a Citizen Science approach, the following steps were taken:

1. Dialogues with the workers;
2. Involvement of the management;
3. Collecting information and help from experts;
4. Providing feedback to the workers;
5. Developing a test phase.

In the first step, semi-structured interviews with workers were conducted to identify barriers, facilitators and useful elements for the development and implementation of a WHPP. Ideas for elements of the intervention and how the intervention could be implemented in the company were generated together with the researchers.

In the second step, as a follow-up to the dialogues with the workers, ideas were discussed and examined on the practical feasibility and achievability with the managers, HR advisors and the prevention team.

In the third step, consultation took place with experts in the field to further develop elements, materials and intervention strategies. This group of experts consisted of experienced researchers in occupational health, experts or trainers in low health literacy communication, experts in the implementation of Citizen Science, and experts from the national occupational health institute of the construction industry.

In the fourth step, the outcomes of previous steps were discussed with workers who were involved from step 1 and those willing to participate at this point of the process. Attention was paid to the proposed content of Citizen Science, the outcomes of other stakeholders, the materials and the intended strategy.

In the fifth and last step, the health promotion interventions were tested and assessed on the feasibility and fit by some workers of both companies, including those involved as citizen scientists from the beginning and other workers. Feedback from the test phase was used to optimize, define and subsequently apply the WHPP within the company.

### Resulting WHPPs: implemented intervention in the construction company and the terminal company

Based on the obtained insights into the barriers and facilitators, three elements were identified with data from the interviews and focus groups as important applying Citizen Science to improve health at the workplace: 1. knowledge and skills, 2. social support and social culture, and 3. awareness about lifestyle behaviors. The strategies to implement these elements as a WHPP were aligned with the company’s specific barriers, possibilities and facilitators [28].

For the construction company, three toolbox meetings covering different health subjects were created with a focus on the improvement of knowledge and skills regarding physical activity at work and during leisure time, nutrition (balance and reading food labels) and goal setting. As toolbox meetings are an existing tool within the sector and company, this felt comfortable and applicable for the target group to use as an intervention strategy. Toolbox meetings consisted of 30-minute interactive sessions, in which workers discussed these topics and shared their own experiences. Furthermore, ideas were shared during the meetings and an idea box was provided at each workplace to collect health-related ideas of workers.

The intervention at the terminal company consisted of two interactive workshops which were introduced as the Tip Top Fit intervention, with a focus on practical information and tips concerning health. During the first workshop, information was provided about lifestyle, health and factors that might influence health at the workplace. Furthermore, workers were provided with health behavior tools, including a food diary and pedometer. During the weeks after the first workshops, workers examined their lifestyle with the use of these tools. The second workshop focused on discussing the workers’ findings after workshop 1. There was also room for discussing individual goals and organizational suggestions for improvement.

### Data collection

Data were collected by questionnaires, interviews, logbooks and a poll. Workers were asked to complete a questionnaire, comprising of questions regarding evaluation elements, at baseline and after the intervention implementation. This evaluation included only the questionnaire data provided by the construction company. Questionnaire data from the terminal company was namely not suitable for evaluation due to a low response rate on both measurements and incomplete information (responses). At T1, a total of four questionnaires were completed by workers of the terminal company. In addition, data on the evaluation questions in these questionnaires was missing and therefore not included for analysis. After the intervention period workers of both companies were asked to participate in interviews to evaluate the intervention. Both workers that participated as citizen scientist, and workers that were not involved as citizen scientists were asked to participate. In addition, paired interviews were conducted with the management and HR-advisors to evaluate the experiences regarding the process and the implemented intervention. Logbooks were kept by researchers to monitor the process throughout the study period. At the terminal company workers were asked to give their opinion regarding the best ideas of the Tip Top Fit intervention. Workers were asked to rate four ideas through a poll with a score ranging from 1 to 4, with a high score indicating the best idea. This Tip Top Fit poll was organized at the end of the intervention period for the workers of the terminal company.

**Fig. 1:**
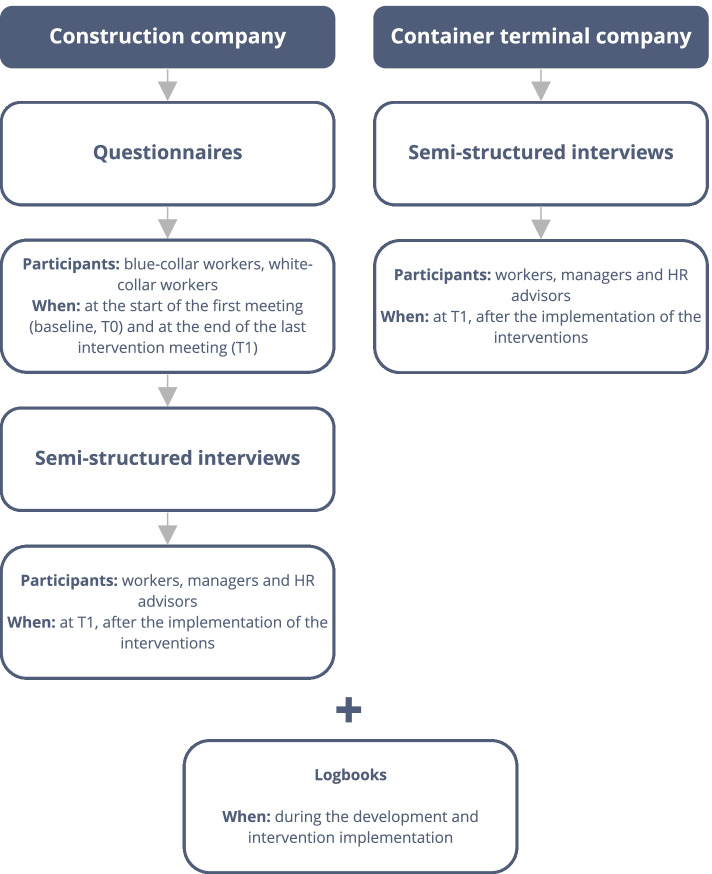
Process evaluation steps of the Citizen Science Approach and the resulting WHPPs

To evaluate the process of Citizen Science and the resulting interventions, questionnaires were handed out and filled in at the start of the first meeting (baseline, T0) and at the end of the last intervention meeting (T1). At the construction company, 175 workers filled in the questionnaire at T1. A group of 133 workers filled in the questionnaire at T0 and T1 and was included for the analysis of the readiness for change. The questionnaire at T0 and T1 comprised validated questions related to health and lifestyle. The questionnaire at T1 comprised additional evaluation questions that covered the following process components of the Nielsen and Randall framework: participation, reach, satisfaction, tailoring, exposure, culture, readiness for change, and perception [29–31]. These evaluation questions, included in the questionnaire at T1, were analyzed for this process evaluation. Workers were asked to reflect on evaluation statements that referred to the experienced involvement, room to discuss opinions, connection of the intervention with desired health, the alignment of ideas to needs and wishes, the experienced health changes, and the perceived support from colleagues and employer. The level of agreement with each statement was answered with a 5-Point Likert Scale. Finally, the intervention could be rated with a grade ranging from 1 to 10, with higher grades indicating higher perceived satisfaction. An overview of the evaluation process is visualized in fig. 1.

After the intervention period, individual face-to-face semi-structured interviews and paired semi-structured interviews were conducted among workers, managers and HR advisors of both companies. A mix of citizen scientists and workers who were not involved as citizen scientist were included to collect different perspectives on the intervention. In total, 13 interviews took place, of which seven at the construction company and six interviews at the terminal company. In total 17 participants were included in the interviews. For the analyzation process, the interviews were recorded with consent of the interviewees. The duration of the interviews varied between 20 to 50 minutes. Interview topics, covering process components of the Nielsen and Randal process evaluation framework, were predetermined and described in an interview guide [29].

During the study period logbooks were kept to record the progress, observe results and to collect notes. These logbooks, including field notes, were maintained by the principal researcher. Two separate logbooks were kept in Word documents to monitor the pilot phase and the implementation of interventions at both companies. The notes were organized by date to easily link the logbook information to the moment in the study process.

### Process measure and evaluation components

To evaluate the intervention process, the framework of Nielsen and Randall was used [29]. This framework focuses on organizational workplace interventions and describes process components that could have influence on the intervention results. The evaluation of process components was divided into three main themes: the intervention design and implementation, the context and mental models. These main themes were supported with accompanying process components for evaluation. The intervention design and implementation theme focused on the intervention exposure, activities and the strategy for the intervention implementation. Process components within the theme of intervention design and implementation were: communication, participation, reach, satisfaction, tailoring, exposure and participation/utility. The context theme consisted of factors that hindered or facilitated the intervention implementation and was evaluated on the process components of culture and events. The theme of mental models provided insights into the perceptions and behaviors of the study population with process components of readiness for change and perception. An overview of all three themes with accompanying process components, definition, operationalization, data collection instrument(s) and measurement moment is presented in Table 1. In this table the process components are defined and operationalized with the use of questions. In addition, each process component contains information about the data collection instruments that are used to collect information and information about the moment of measurement.

**Table 1:** Intervention design and implementation, Context and Mental models supported with process component, definition description, operationalization, data collection instrument and measurement moment

Framework component	Definition	Operationalization	Data collection instrument	Measurement moment	Evaluation component(s)
I. Intervention design and implementation					
*Communication*	Communication and awareness about the intervention	Q; How was the intervention/action plan communicated to all workers at the start? Q; Did you know beforehand that you receive a toolbox regarding health and what was included? Q; How was the intervention communicated to the workers at the start?/ How were participants informed about the intervention? Q; What communication tools were used to communicate the intervention to the workers?	Interviews Logbooks	T1 T0 - T1	Citizen Science approach, resulting WHPPs
*Participation*	Participative role and experience of involvement in the intervention	Q; How did workers participate as Citizen Scientists and did they feel responsible for the intervention? Q; Did the workers felt involved during the intervention? (assisting/participating with thinking about improvements) Q; Citizen Scientists: Did you actively participate as motivator/ health ambassador?	Interviews Questionnaire Logbooks	T1 T1 T0 - T1	Citizen Science approach, resulting WHPPs
*Reach*	Level of attendance and participation of target audience	Q; How much workers were reached with the Citizen Science approach?	Interviews Questionnaire Logbooks	T1 T0 T0 - T1	Citizen Science approach, resulting WHPPs
*Satisfaction*	Satisfaction with the intervention strategy (participatory approach)	Q; Appropriateness of tools and materials of the CS approach including workshop tools and intervention activities chosen. (parts of approach that were the most valuable) Q; Were management and the participated workers satisfied with the CS approach? (suggestions for possible improvements) Q; What is your opinion about the intervention/toolbox?	Interviews Questionnaire Poll Logbooks	T1 T1 T1 T0 - T1	Citizen Science approach, resulting WHPPs
*Tailoring*	Target fit with workplace problems and needs	Q; Did the interventions target the right problems/health issues at the workplace? Q; Do the interventions fit with the needs and the workplace? Q; To which extend were the approach and the intervention(s) tailored for target group?	Interviews Questionnaire Logbooks	T1 T1 T0 - T1	Resulting WHPPs
*Exposure*	Exposure to implemented intervention elements	Q; Were the workers exposed to the interventions that were implemented at the workplace? Q; Did the workers notice changes at the workplace and what kind of changes regarding health they noticed. Q; Did they notice that ideas are implemented in order to make the workplace healthier?	Interviews Questionnaire Logbooks	T1 T1 T0 - T1	Resulting WHPPs
*Participation/Utility*	Use of intervention implementations	Q; Do you use the implemented interventions?	Interviews Logbooks	T1 T0 - T1	Resulting WHPPs
II. Context					
*Culture*	Organizational/workplace culture influence on the facilitation and implementation of the intervention	Q; Why were intended CS elements or interventions not implemented Q; Why were some ideas selected for implementation and why not? Q; How did the intervention fit with the culture and conditions of the intervention group?	Interviews Questionnaire Logbooks	T1 T0, T1 T0 - T1	Citizen Science approach, resulting WHPPs
*Events*	Interfering events with possible influence on the implementation and intervention process	Q; Did any events took place that might interfere with/influenced the intervention implementation? Q; Did changes outside the organization take place during the intervention phase? (legislation, recession etc.)	Interviews Logbooks	T1 T0 - T1	Citizen Science approach
III. Mental models					
*Readiness for change*	Extent to which the target group was ready for change	Q; Was the target group ready to change/motivated to change their lifestyle? Q; Were you ready to execute and use the implemented intervention plans? Q; Did you feel ready to execute and use the implemented interventions at the workplace?	Interviews Questionnaire Logbooks	T1 T0, T1 T0 - T1	Resulting WHPPs
*Perception*	Perception of intervention (positive or not)	Q; How did the workers and the management perceive the implemented interventions (positive or not)? Q; Do the workers think that the workplace changed positively? Q; What did the changes on the workplace mean for you/your colleagues?	Interviews Questionnaire Logbooks	T1 T1 T0 - T1	Resulting WHPPs

For the evaluation of the Citizen Science approach, communication, participation, satisfaction, culture and events were included as process components. The process components: communication, participation, reach, satisfaction, tailoring, exposure, participation/utility, culture, readiness for change and perception were included to evaluate the resulting WHPPs.

The Intervention design and implementation involved the following framework components: communication, participation, reach, satisfaction, tailoring, exposure, and participation/utility. Communication was operationalized as the adequacy of information and communication about the intervention, participation involved the experienced degree to which the workers felt involved during the process and intervention and reach was defined as the level of attendance and participation of the target audience. Further, satisfaction was operationalized as the satisfaction with the intervention strategy, including the workers’ opinion regarding the appropriateness of tools and materials, intervention activities and intervention approach. Tailoring involved the degree to which the intervention was suited to the workplace’s needs and health problems and fits the target group.

The last element included participation or utility, which referred to the extent to which the interventions were actually used by the workers.

The context involved the components culture and events. Culture was operationalized as company characteristics and relations that could have influence on the facilitation and implementation of the Citizen Science approach and intervention. Events was defined as possible interfering occurrences that could influence the implementation and intervention process.

The third theme, mental models focused on the behavioral aspects that could be driven by perceptions and readiness for change of the target population. Perceptions involved perceived change as to health promotion at the workplace the influence of the workplace on the workers’ health, while readiness for change comprises the readiness and motivation of the target group to change their lifestyle and apply intervention elements.

### Data analysis

Quantitative data, collected with questionnaires, were analyzed using the IBM statistical software program SPSS version 22. Statistical analyses consisted of descriptive statics, including frequencies, percentages and mean scores. Participants were included in data analysis if they filled in the evaluation questions from the questionnaire at T1.

Audio recordings of all interviews were transcribed and analyzed using the qualitative research software MAXQDA. Qualitative content analysis according to the methodological approach of Kuckartz was applied to analyze the interview data [32]. This method entails different steps for analyzing qualitative data, including the identification of patterns, summarizing codes, and mapping results according to the framework of Nielsen and Randall. To begin with, all transcripts were read to become familiar with the data. Repeated patterns were identified and summarized as codes and organized into subthemes according the process components of the framework of Nielsen and Randall. To ensure reliability and consistency of the coding process, the first two transcripts were independently marked and coded by the first and second author (LL and SvdF). The codes were compared and discussed to ensure correct classification and identification of subthemes based on the elements of the framework of Nielsen and Randall and the principles of thematic content analysis [29, 33]. Interview quotations were translated from Dutch to English.

## Results

Two events during the study period influenced the Citizen Science approach and interventions implementation. These events included organizational changes at the terminal company and the Covid-19 pandemic. As a result, interventions were postponed because organization possibilities for intervention implementation were limited and some workers and health ambassadors dropped out.

### Evaluation citizen science approach

Findings are reported according to the Nielsen and Randall framework, structured by three themes with accompanying process components (see Table 1). For the evaluation of the Citizen Science approach, the following two of the three evaluation themes were evaluated: intervention design and implementation, and context. Process components that were included in these themes and structured the study results consisted of: communication, participation, satisfaction, culture and events were included as process components for the evaluation of the Citizen Science approach.

#### Intervention design and implementation

At the construction company, workers were approached and informed by their managers. At the terminal company acquaintance with the intervention and the registration to participate was organized via sessions and through e-mail contact. Based on the interviews, some workers of the terminal company experienced that the overall communication went well. For example, one worker of the terminal company said: *“yes, I definitely felt involved in the emails, and overall the communication went well.”*

However, in both companies, some workers mentioned that the communication intensity decreased during the course of the intervention and the preference for continued communication. As one worker said: *“I least liked the attention for it, because at a certain moment there was no communication.”* Also, a need for a clearer introduction and information provision to the managers at the beginning of the intervention was mentioned, as one worker of the terminal company, for example, suggested: *“We should have organized a meeting for the managers. That is something we should adapt in the future.”*

According to the interviews, the majority of the interviewed workers at both companies indicated that they were actively involved in the intervention. One worker of the construction company, for example, reported: *“I certainly felt involved, but that’s because it’s a subject that interests me.”* Another worker said: *“Yes, I did felt involved. But, as I said, I am into the subject around nutrition, so then it interests you.”* Workers of the terminal company suggested that the involvement of managers could be optimized by organizing an information session for the managers.

As to the participation as health ambassador, some workers experienced to have little influence, and some workers mentioned that they actively motivated colleagues and promoted the intervention to colleagues. It was also expected that the impact of health ambassadors could be greater. As one worker of the terminal company reported: *“Perhaps the expectation that they would start small projects.”* Another worker added: *“I also expected more from the ambassadors, that it would become more alive and that they would manage to involve more people.”*

Interviewees experienced the involvement in content creation and the approach as positive and the open atmosphere created enough opportunities for questions. As one worker of the terminal company reported: *“I liked it the most to be involved in the beginning and during the conversations to find out what should be included in the intervention. There was more room for conversations, more people were present and I was able to share my ideas.”*

#### Context

According to interviewees of the construction company, their company is open-minded, which offers room for opinions from workers. Besides, most workers indicated that they felt supported by their employer. Although workers at the terminal company indicated that they have a pleasant culture at the workplace, they indicated the need for more guidance and involvement from the employer/supervisor.

Furthermore, workers mentioned in the interview that the employer should emphasize the importance of participation in the intervention with special attention for the workers’ health. One worker, for example, expressed this opinion as: *“It must become healthier, but not only to reduce absenteeism. Then people would start to think that it is only in favor of the employer.”*

Some differences as to the culture among older and younger workers seemed to appear from the interviews. According to the interviewees, the older generation within the companies are less concerned about their health, compared to the younger generation of workers.

Workers in both companies mentioned in the interview that the mentality is characterized by an ‘alpha’ culture with dominant male leadership and an individualistic attitude. In addition, workers of the terminal company indicated that there is a conservative and distrustful attitude, which negatively influences the willingness. One worker said: *“We have been used to something for years and when something changes, it is always an issue. The fear of changing something.”* Besides, workers do not always feel comfortable to talk about personal topics such as health.

According to some workers, physical work-related factors and a high workload might have influenced the intervention implementation to some extent. For example, one worker of the construction company said: *“The pressure is quite high. You also have to deal with deadlines.”*

While some workers at the construction company were more aware about their health due to the pandemic, others indicated that they were eating less healthily and were struggling to be physically active. For example, one worker reported: *“We talk more often about health, especially now during corona you are more aware of it.”* Another worker indicated: *“I notice that I became an emotional eater during that time.”*

During the study period there were organizational changes that led to the drop out of workers and ambassadors and delay in the implementation of the intervention. According to the logs, it was difficult to schedule workshops and training courses. Moreover, another big ‘event’ that influenced the intervention process and approach was the Covid-19 pandemic. According to the logs, this led to a postponement of the intervention and limitation of the organization’s possibilities.

### Evaluation resulting WHPPs

For the evaluation of the Citizen Science approach, the following three evaluation themes were evaluated: intervention design and implementation, context, and mental models. To evaluate the study results of the Citizen Science approach according to these themes, the following process components were included: communication, participation, reach, satisfaction, tailoring, exposure, participation/utility, culture, readiness for change and perception were included as process components for the evaluation of the resulting WHPPs. An overview of the three evaluation themes with accompanying process components and operationalization are presented in Table 1.

#### Intervention design and implementation

Some workers of the construction company reported in the interview they obtained some prior knowledge about the toolbox before the start, which involved mainly information about the toolbox in general and the included topics of the toolbox. Interviewees who indicated that they had no prior knowledge about the intervention, indicated that they started their intervention with no specific expectations.

Questionnaire data showed that a majority of the workers who had filled in the questionnaire (62%, *n* = 109/175) felt involved during toolboxes. With regard to the statement about the probability of expressing an opinion during sessions, 72% (*n =* 126/175) of the participants at the construction company agreed.

With regard to the participation, it was mentioned in the interview that involvement was especially experienced at the beginning of the intervention. According to the logs of the terminal company, during meetings with workers and employers about the approach of the intervention, there was little interaction with and input from the workers. As communicated by a worker, most workers did not feel comfortable having the meeting together with the employer/management. In contrast to the terminal company, logs of the construction workers indicated a positive atmosphere with a lot of interaction during toolbox sessions, especially in small groups. During sessions with larger groups, more resistance was felt.

According to the interviewees and the logs, improvement of the participation and involvement throughout the intervention mainly consisted of the advice to narrow the group sizes. One worker of the construction company said: *“In a group it is just striking that some do not dare to ask questions, they are afraid that they will be laughed at. It would be better to organize it one on one or in small groups.”*

From the questionnaire data, it appeared that about 83% of the workers who filled in the questionnaire (*n =* 151/175) indicated that they were present at toolbox 1 and 77% (*n =* 135/175) indicated that they were present at toolbox 2.

Workers of both companies agreed upon the fact that it is not feasible to reach everyone with the intervention, since it does not fit everyone’s interests. The older generation in particular was mentioned as difficult to reach, as one worker of the terminal company reported: *“The older generation thinks very differently. They are much less concerned about their health. They don’t take it seriously.”*

Moreover, planning outside working hours was experienced as a pressure on flexibility. As described in the logs, the management of the terminal company had decided that the workshops had to be followed in the workers’ own time because it was considered impossible for the company to give the workers time off to follow the workshops. According to workers of the terminal company, the relatively low attendance was probably due to a misfit with interests and the unfavorable planning. As indicated in the logs of both companies, not all workers were able to attend sessions because, for example, they had to work changing shifts or because they were following another course at that time.

Workers of the construction company indicated that they appreciated the non-committal nature of participation. In contrast, workers of the terminal company indicated that not making the session mandatory may have affected the reach negatively. One worker of the terminal company said: *“It would be a very good way to oblige, because this is optional and the toolbox is mandatory.”*

Satisfaction with the toolbox health was rated with a mean score of 6.7 (SD = 1.4). A minority of 14% (*n =* 25/175) of the workers rated the satisfaction as insufficient and scored it with a 5 or lower. According to the interviews, overall, the toolbox was experienced as fun and interesting, being a good initiative and concept to draw more attention to health in the workplace. One worker terminal company said: *“I think the positive aspect about the toolbox is that you try to raise some awareness about health.”* Another worker reported: *“For me it was like a reset.”*

From the poll about the ideas of Tip Top Fit intervention within the terminal company, the idea of presenting flyers with tips and tricks about nutrition/sleep/work during night shifts was perceived as the best idea with a total score of 265 (29%, *n =* 265/902). With a score of 223 points (25%, *n =* 223/902) the idea of health information provision became second, followed up by the idea of using stickers to visualize healthy food choices with 219 points (24%, *n =* 219/902), and the idea to encourage to take the stairs instead of the elevator with 195 points (22%, *n =* 195/902).

As to the appropriateness of tools and materials, some workers reported that they experienced the use of the pedometer and the food diary useful, others indicated that they used them not often or not at all. Moreover, it was mentioned that a one-hour session may not be functional enough and that there was a need for structural repetition.

Most workers agreed that they would recommend the intervention for other companies. One worker of the construction company, for example, said: *“In this particular branch of construction work it is very difficult to eat really healthy, but I do think this toolbox is advisable for other companies.”*

Based on the questionnaire, just over half of the workers at the construction company agreed on the statement concerning the alignment of ideas with individual wishes to make the workplace healthier (54%, *n =* 94/175). Moreover, a small majority of the participants (51%, *n =* 90/175) agreed on the statement “The health toolbox matches the wishes I have with regard to my own health”; 36% (*n =* 63/175) neither agreed nor disagreed and 11% (*n =* 20/175) disagreed with the statement.

As to the perceived intervention fit with the problems and needs of the workplace, some workers of the construction company mentioned that the intervention did not match the interests and needs of some of the construction personnel. One worker reported: *“As I mentioned, we get enough daily exercise on a day of work.”* Another worker said: *“I think office workers would benefit more from it, because they have a sedentary profession.”* Some felt it was not suited for their organization because it was not in balance with the high workload. For example, one worker said: *“The pressure is quite high. You also have to deal with deadlines.”* As noted in the logs, there was uncertainty as to whether the approach would match the target group.

In contrast, some workers of the terminal company reported that the intervention was well suited to their interests and work environment. One worker reported: *“I thought it was useful. Especially the information regarding night shifts, what you can and cannot eat and how the biological clock works. Now I am aware of what to eat when I’m feeling tired.”*

About half of the workers working at the construction company (49%, *n =* 86/175) neither agreed nor disagreed with the statement regarding the perceived health change. About 28% (*n =* 49/175) agreed that the workplace has become healthier, while 18% (*n =* 31/175) disagreed on the statement.

While some workers of both companies experienced health improvements, others mentioned they noticed little or no change at the workplace. One worker of the construction company reported: *“I don’t think something changed yet. We’ve had the toolbox, but I don’t think people are working on it yet.”* Another worker said: *“I don’t think you can change that with a toolbox, that’s very difficult I think.”*

Some workers at the construction company mentioned that they were not aware of the existence of an idea box nor the implementation of the ideas. One worker reported: *“At least I haven’t seen an idea box.”* Another worker said: *“I think that idea box was used minimally.”* A possible reason for the minimal use of the supplied ideas box may be related to the fact that the construction company already had a general idea box.

With regard to the use of implemented interventions, some of the workers at both companies indicated in the interviews that they had made some positive adjustments. One worker of the terminal company said: *“I did start with fitness and also started living healthier. At least, I try.”* According to some workers of the terminal company, the information and shared experiences were very helpful to make healthy improvements in their diet. Some mentioned that they eat more consciously and healthfully during night shifts and that they exercise more often.

At the construction company, a number of workers mentioned that they stopped eating more fruit and did not use the idea box anymore. In contrast, others indicated that they try to eat healthier, exercise more and snack less unhealthy. One worker said: *“Eating healthier, you try to think about that more often.”*

#### Context

About half of the workers at the construction company (49%, *n =* 86/175) neither agreed nor disagreed on the statement about the experience that it was easier to talk about health with colleagues after the toolbox. With 22% (*n =* 38/175), the number of workers who agreed was almost equal to the number of workers who disagreed on this statement (21%, *n =* 37/175). Also, mixed results appeared on the perceived support from colleagues as well as from the employer.

#### Mental models

A majority of 52% (*n =* 70/133) (T0) and 51% (*n =* 68/133) (T1) indicated that they were already living a healthy life. In addition, a percentage of 38% (*n =* 51/133) who intended to live healthier at T0, remained unchanged at T1.

Some workers were satisfied with their current lifestyle and therefore felt no need to change. One worker of the construction company said: *“We are physically active enough already. I walk about 12,000 steps a day.”* Another worker of the construction company reported: *“I’ve just lived the same as I always live, I’m not going to change anything. Because I know that I eat healthy, eat enough vegetables and enough fruit, sometimes even too much.”*

Data from the questionnaire underlines the influence of the workplace’s health on workers. While 31% (*n =* 54/175) neither agreed nor disagreed on the statement “It helps me personally if the workplace becomes healthier”, a total of 54% (*n =* 95/175) agreed to strongly agreed on the statement (47%, *n =* 83/175; 7%, *n =* 13/175).

## Discussion

The current study evaluated the process and experiences of applying a Citizen Science approach to co-create and implement WHPPs. The study findings emphasize the importance of sustained engagement over time, paying attention to the communication and involvement throughout the entire development and implementation process. Besides the workers’ involvement, it appeared that there was a need for more guidance and involvement from the employer and managers. According to the workers this could have been optimized with better information provision. Moreover, it appeared that a misalignment with the interests of all workers and the unfavorable planning outside working hours would have negatively influenced the willingness to participate and the reach. Continuous information provision, sustained engagement over time, better alignment with the workplace’s culture were considered to be important improvements for optimal involvement and reach. Finally, factors that influenced the approach were the social culture, including generation differences in willingness and interests, and interfering events, such as high workload, organizational changes, and the unforeseen Covid-19 pandemic.

With regard to the evaluation of the resulting WHPPs, the toolbox was moderate positively experienced among workers of the construction company. Smaller groups might positively influence the feeling of openness among workers, which could lead to more involvement and interaction during the sessions. Furthermore, the unfavorable planning outside working hours was perceived as a pressure on flexibility and affected the reach. Workers indicated the need for structural repetition. One-hour sessions may not be functional enough to achieve the desired health improvement. With regard to health improvement at the workplace, experiences varied from recognizing some health improvement to temporary change or experiencing no change. Looking at the readiness for change, the intention to live healthier remained unchanged at both companies, whereas the general health perception was improved after the intervention period.

The results of this process evaluation indicate that the implementation of WHPPs in this particular setting requires sustained investment in motivating and engaging workers. The environment seems to play an important role in the engagement of workers. Workers indicated the need for more guidance and involvement from the employer and managers through proper information provision and favorable planning. These results seem to be consistent with other research, which found that support and commitment from the company, including management, is vital for effective implementation of health interventions [34, 35]. Providing the ability to actively participate in the intervention through favorable planning is considered to be an important element of organizational support. In the construction industry, this often seems to be difficult to realize because of the target driven-culture, which makes the management more reluctant to allocate time for participation in interventions [36]. The previous study already showed that the participative approach, including the role of citizen scientist, would be challenging to implement due to the workload and lack of time [28]. Results from the current process evaluation underline this challenge and indicate that possibly more organizational support through favorable planning could improve the engagement and reach. The lack of time among workers is important in the implementation of a Citizen Science approach, since this could have resulted in less participation and involvement of workers and more guidance from the researchers [28].

Organizational changes at the terminal company and the Covid-19 pandemic at the construction company influenced the planned co-created intervention implementation. The organization’s social culture seems to be another factor that holds an important role in effective implementation of a WHPP with Citizen Science approach. Aside from the generation differences, not all workers were interested in or open for improving their health. Besides, health appeared to be considered as a personal topic and was therefore often not discussed at the workplace. These findings are in line with previous research among blue-collar (construction) workers showing that the social culture, characterized by the masculinity, could negatively influence the health behavior and acceptance of WHPPs [37, 38]. Paying attention to the improvement of social support and culture can therefore be regarded as an important starting point for the implementation process.

With regard to the intervention strategy and the intended health improvement, results emphasize the importance of structural repetition. As was experienced by the workers, a more intensive approach with longer time span would be needed to enable improvement of the health of blue-collar workers at the workplace. One-hour sessions with health information were regarded as not functional enough and workers indicated that the attention to live healthier decreased over time. According to previous research, the establishment of a culture in which sustained health improvement is supported and a healthy lifestyle becomes a standard, is regarded as essential element that requires long term efforts [13, 39].

The current study provides insights into the implementation and experiences of a Citizen Science approach and the resulting WHPPs to promote blue-collar workers’ health in two companies. Study results showed that overall, the workers were satisfied with the experienced involvement, especially at the beginning of the intervention. However, results also showed that organizational and social cultural factors created hindering conditions for the Citizen Science approach and the implementation of the health promotion interventions. These factors, which can be classified into contextual and personal factors, were identified during the preceding Citizen Science steps as possible barriers for Citizen Science to improve health at the workplace [28], such as a high work pressure, lack of time, lack of social support, masculine culture, and a negative attitude.

Results of this evaluation showed that the high work pressure and lack of time together with unfavorable planning outside working hours made it difficult to participate or be actively involved as health ambassador. As to the lack of social support and the masculine and negative culture, this would have negatively influenced the willingness to participate as well as the reach and involvement. Suggested by the workers, the involvement of the employer and management could be improved by organizing information sessions for the employer and management. Moreover, support from the employer and management also includes finding ways to incorporate the intervention into the work schedules and thereby limit the pressure on flexibility of the workers. For example, the intervention can be included as part of existing and planned mandatory sessions, so that the intervention sessions would not have been organized in the workers’ spare time. With regard to the social culture, results underline the importance of creating an open environment in which health can be discussed. Taking into account the present masculine culture and the negative attitude, it seems important to organize the interventions in small groups. According to the logs and the feedback of workers, small groups are preferred and would positively influence the feeling of openness among workers, which could lead to more involvement and interaction. Overall, the current results show the importance of taking contextual and personal factors into account.

The obtained insights also showed possibilities and the ability of using a Citizen Science approach to improve health in occupational settings with blue-collar workers. The overall satisfaction and involvement of the approach and interventions was positively experienced by the workers. However, since barriers and facilitators differ in organizational settings, further research would be valuable to evaluate how and to what extent a Citizen Science approach could be successfully applied to co-create and implement health interventions to promote health in other occupational settings.

Moreover, this evaluation shows that despite aligning the strategies with the company’s identified barriers, in practice it may not always be possible to actually tackle these barriers. Therefore, continuous monitoring could be helpful in order to anticipate on external factors and increase the adaptability of the approach and implementation. For future studies it may be valuable to view the evaluation as a dynamic process. For example, the Dynamic Integrated Evaluation Model developed by Von Thiele Schwarz et al. [40] integrates the evaluation throughout the intervention. Herewith, the evaluation is not conducted afterwards, but considered as an iterative process that continuously provides insights, allows for adjustments during the process, which also strengthens the co-creation.

One strength of the current study is the use of quantitative and qualitative data. The use of different data sources contributed to a thorough evaluation of the experiences and the process of development and implementation. Qualitative data and information from the logbooks provided in-depth details and nuances that complemented the questionnaire results. Also, the use of the theoretical framework from Nielsen and Randall could be regarded as a strength. Process components of the framework provided a structured approach to the evaluation. This approach made it possible to highlight different perspectives based on the extensive and detailed evaluation of the intervention design and implementation, the context and mental models.

There were also some limitations in the current process evaluation. The framework of Nielsen and Randall was used to evaluate both the Citizen Science approach as well as the resulting interventions. However, for some components of the framework, including the component satisfaction, more information was collected aimed at the evaluation of the interventions. As a result, it was not possible to include all components of the framework for the evaluation of the Citizen Science approach. Furthermore, because of the low response and incomplete questionnaire data availability at the terminal company, questionnaire data could be analyzed from the construction company only. Aside from the fact that this could somewhat have limited the generalizability of the results, the quantitative evaluation data could not be compared between the two companies. Due to the missing questionnaire results, we were also unable to cross verify the findings with the qualitative results.

In addition, the representativeness may also be influenced by the possibility that workers who were positive minded towards the intervention, were more likely to fill in the questionnaires and participate in the interviews. However, with the use of different perspectives from citizen scientists and workers together with different data sources, including interviews, questionnaires and logbooks, we were able to cross verify results and reduce the risk of biased study findings and conclusions.

Another limitation is the difference in the type of information to evaluate the Citizen Science process and the resulting interventions equivalent. For the evaluation of both interventions and the Citizen Science process, the aim was to collect the same type of information. However, due to a major reorganization and the covid-19 pandemic, this was not feasible within the terminal company resulting in too few questionnaire data. Thus, more information was obtained from the construction company. As there was too little data available from the terminal company to compare the results side by side, the results have been combined and the striking differences between the two companies have been indicated when this was the case.

Finally, another limitation of the present study is the lack of documentation on the exact distribution of the number of blue-collar and white-collar workers who participated in the interviews and questionnaires. As this missing information hinders the method’s reproducibility, we must conclude that this is a deficiency of our study. Nevertheless, because this study focuses only on the process and not on the effectiveness, we think that the impact on the results is acceptable and that the results give a useful impression of the citizen science approach for the implementation of a worksite health promotion program for blue collar workers.

## Conclusions

The first study objective focused on the evaluation of the experienced Citizen Science approach. Overall, satisfaction and involvement of the approach and interventions was positively experienced by the workers, especially at the beginning. Organizational and social cultural factors created hindering conditions for the Citizen Science approach and the implementation of the health promotion interventions. Continuous information provision and engagement over time, better alignment with the workplace’s culture and favorable planning were considered to be important factors for facilitating involvement, reach and satisfaction of the workers in a Citizen science approach to design and implement a WHPP. With regard to the second study objective about the worker’s experiences of the implemented WHPPs, involvement and interaction were particularly experienced in small grouped sessions and one-hour sessions were perceived as not functional. Workers indicated a need for structural repetition. The addressed improvements with regard to contextual and personal factors need be considered when designing and implementing WHPPs with Citizen Science approach among blue-collar workers. Besides, more studies evaluating the process of WHPPs with Citizen Science approach among blue-collar workers could provide further insights into the implementation process and successfulness of the Citizen Science approach.

## Data Availability

The datasets generated during and/or analyzed during the current study are not publicly available due to privacy motives of the participants, but are available from the corresponding author on reasonable request.

## Abbreviations

WHPPs: Workplace Health Promotion Program
CS: Citizen Science
T1: Baseline measurement
T2: Follow-up measurement
